# Understanding how social support influences peer-delivered HIV prevention interventions among Ugandan female sex workers: a case study from HIV self-testing

**DOI:** 10.1186/s12889-022-12836-3

**Published:** 2022-03-03

**Authors:** Maureen McGowan, Stephanie D. Roche, Aidah Nakitende, Jonas Wachinger, Esther Nanyiri, Jocelyn Amongin, Ajiri Nakabuye, Daniel Kibuuka Musoke, Shannon A. McMahon, Till Bӓrnighausen, Katrina F. Ortblad

**Affiliations:** 1 Heidelberg Institute of Global Health, Heidelberg University, Im Neuenheimer Feld 130.3, 69120 Heidelberg, Germany; 2 Public Health Sciences Division, Fred Hutchinson Cancer Research Center, Seattle, USA; 3 International Research Consortium, Kampala, Uganda; 4 Social and Behavioral Interventions Program, Department of International Health, Johns Hopkins Bloomberg School of Public Health, Baltimore, USA; 5 Department of Global Health and Population, Harvard T.H. Chan School of Public Health, Boston, USA; 6 Africa Health Research Institute, Somkhele and Durban, South Africa

**Keywords:** Sub-Saharan Africa, Peer delivery, Female sex worker, HIV self-testing, Social support

## Abstract

**Background:**

Female sex workers (FSWs) have tightly connected peer networks and remain at high risk of HIV acquisition. Peer delivery of HIV prevention interventions, such as HIV self-testing (HIVST), is a recommended implementation strategy for increasing intervention uptake and continuation among FSWs. We analyzed qualitative data from a peer-delivered HIVST intervention among FSWs in urban Uganda to understand the ways social support within this peer network can motivate or discourage the uptake of peer-delivered HIVST.

**Methods:**

Between February and April 2017, we conducted in-depth interviews (IDIs) with FSWs (*n* = 30) and focus group discussions (FGDs) with FSW peer educators (PEs, *n* = 5) finishing participation in a four-month randomized implementation trial testing models of peer-delivered HIVST in Kampala. FSW participants were ≥ 18 years old, self-reported exchanging sex for money or goods (past month) and had not recently tested for HIV (past 3 months). FSW PEs either directly distributed HIVST kits to participants or provided coupons exchangeable for HIVST kits from specified healthcare facilities. In the IDIs and FGDs, we asked participants to share their experiences receiving or delivering peer-delivered HIVST, respectively. Using a hybrid deductive and inductive coding approach, we arranged findings along the dimensions of an established social support theory: informational, instrumental, and emotional support.

**Results:**

The median age of participants was 30 years (IQR: 27–33) and PEs was 33 years (IQR: 29–37). We found that social support within FSW peer networks both motivated and discouraged uptake of peer-delivered HIVST. For example, sharing positive HIVST experiences (informational support), directly delivering HIVST kits (instrumental support), and encouraging linkage to care (emotional support) motivated HIVST uptake among FSWs. Conversely, the spread of misinformation (informational support), limited HIVST kit availability fostering mistrust of PEs (instrumental support), and fear of social exclusion following HIV status disclosure (emotional support) discouraged HIVST uptake among FSWs.

**Conclusions:**

In Uganda, social support (e.g., informational, instrumental, and emotional support) among FSW peers can work in ways that both motivate and discourage peer-delivered intervention uptake. Future FSW peer-delivered HIV prevention interventions should be designed around the dimensions of social support within FSW peer networks to maximize initial and repeat intervention delivery and uptake.

## Introduction

Peer delivery of HIV prevention interventions has been proven an effective approach for reaching key populations at high HIV risk in diverse settings [1–4]. In sub-Saharan Africa, nearly 20% of new HIV infections are estimated to be among female sex workers (FSWs) and their clients [5, 6]. FSWs are at increased risk of HIV infection for reasons including engagement with numerous sexual partners, inconsistent condom use, and structural barriers, such as stigmatization by self and healthcare providers, limited schooling, long distances to health facilities, high costs of traveling to healthcare facilities, and criminalization of sex work as well as high levels of alcohol and drug misuse [6–9].

In Kampala, the capital city of Uganda, there are roughly 10,000 FSWs (~ 2% of all women ages 15–59); one in three are estimated to be living with HIV [5, 10–13]. A critical entry point for HIV prevention and treatment services among populations at increased risk is HIV testing [8, 14]. A randomized implementation trial in this setting found that peer-delivered oral-fluid HIV self-testing (HIVST) significantly increased recent and repeat HIV testing among FSWs compared to standard facility-based testing [4]. Additionally, FSWs in this trial described peer-delivered HIVST as desirable because it increased privacy, convenience (including time and cost), sense of control, and confidentiality [15, 16]. Although this model of peer delivery has been found to be appropriate among FSWs in this setting, little is known about how FSW social support structures (e.g., informational, instrumental, and emotional support) influence the delivery and uptake of peer-delivered HIV prevention interventions.

Based on evidence that peer education for HIV prevention is significantly associated with increased HIV knowledge and condom use in a variety of settings [17, 18], we hypothesized that, delivering HIVST via trusted peer educators (themselves established members of the FSW community) would generate social support for—and lead to uptake of—HIVST among FSWs. Specifically, our hypothesis and intervention design were grounded in evidence that the likelihood of uptake of peer-delivered interventions increases when delivered by peer educators (PEs) who are trusted members of the target community [17]. We further based our hypothesis and intervention design on evidence that information delivered by PEs (as opposed to individuals outside of the target community) is often perceived by recipient peers not only as culturally appropriate and easy to understand but also as an open invitation to discuss sensitive topics, such as HIV [17].

In this study, we aimed to understand how FSW social support can influence (i.e., motivate or discourage) the uptake of peer-delivered HIVST in urban Uganda. Specifically, we aimed to understand how FSWs experienced social support within the context of the intervention and how this compared with the intended intervention design. We hope that a better understanding of FSW peer interactions in this setting can inform the design of future peer-delivered HIV prevention interventions among FSWs and other key populations to maximize intervention implementation and outcomes.

## Methods

### Study design

Our study was part of a larger randomized implementation trial that tested the impact of two different models of peer-delivered HIVST on recent and repeat HIV testing among FSWs in Kampala, Uganda. The details of this trial, designed by authors DKM, TB and KFO, are described elsewhere [4]. Briefly, the trial randomized 960 participants to either: 1) direct peer-delivered HIVST kits, 2) peer-delivered coupons exchangeable for HIVST kits at nearby healthcare facilities, or 3) peer-referral to free, facility-based HIV testing services (standard of care). The PEs delivering HIVST kits, coupons, or referrals either had prior experience working as PEs for local nonprofits serving FSW communities (i.e., as PEs of HIV prevention and family planning programs) or were selected by established PEs because they were well-known and trusted within their FSW community. PEs were selected with the assistance of the aforementioned nonprofits and staff from the Uganda’s Most at Risk Population Initiative (MARPI). All PEs completed a one-day training on the intervention and study procedures. Thereafter, PEs recruited eight participants through their social networks. Eligible participants were ≥ 18 years, self-reported exchanging sex for money or goods (in the past month), self-reported being HIV-negative or having an unknown status and had not recently tested for HIV (in the past 3 months). In addition, eligible participants had no prior experience using an oral-fluid HIVST kit.

Over the four-month trial duration, PEs conducted four visits with participants including one group visit (at 0 months) and three one-on-one visits with participants (at 0.5, 1.5, and 3 months). During these visits, PEs provided information on HIVST (intervention arms only), encouraged participants to access facility-based HIV testing and/or confirmatory HIV testing, distributed condoms, and screened for adverse events (e.g., intimate partner violence, mental health distress). At the first and fourth PE visit (months 0 and 3), PEs in the HIVST intervention arms delivered to participants either an HIVST kit or a coupon exchangeable for an HIVST kit at a nearby health facility. We compensated PEs 90,000 Ugandan Shillings (UGX) (~$25 United States Dollars [USD]) for completion of each round of PE visits (for a total of four rounds), which is similar to what other interventions in Uganda have compensated FSW PEs.

We obtained ethical approval for this study from by the Institutional Review Board of the Harvard T. H. Chan School of Public Health (IRB16–0885) and the Mildmay Uganda Research Ethics Committee (REF 0105 ± 2016). All peer educators and participants provided written informed consent for study-related activities and assessments.

### Data collection

At baseline (month 0), all participants completed quantitative assessments which included the collection of socio-demographic data (e.g., age, education, income). At study completion (month 4), 23% of randomly sampled PEs participated in focus group discussions (FGDs) and 5% of randomly sampled participants completed in-depth interviews (IDIs).

#### Focus group discussions

We conducted five FGDs with 28 PEs (5–6 PEs per group, from a mix of study arms) to capture their collective experiences implementing peer-delivered interventions (e.g., HIVST or referral to facility-based HIV testing services) [19]. In our semi-structured FGD guides (developed in collaboration with MARPI), we asked PEs to discuss their experiences with intervention delivery, relationships with participants, and recommendations for future peer-delivered interventions. Ugandan researchers with graduate-level qualitative training (authors A Nakitende, EN, JA, A Nakabuye) conducted the FGDs in a private conference room of a local NGO. We conducted all discussions in Luganda or English, according to the preferences of the PEs in each group. PEs were compensated 16,500 UGX (~$5 USD) for participation in the FGDs.

#### In-depth interviews

We conducted individual IDIs with participants across study arms to understand their experiences receiving peer-delivered HIV prevention interventions [19]. We developed semi-structured interview guides, again in collaboration with MARPI, and pilot tested these guides prior to implementation. The guides sought to understand participants’ social networks as well as their perceptions of and experiences with peer-delivered HIVST or referral to facility-based HIV testing. The same Ugandan researchers described above conducted the IDIs in participants’ preferred language (Luganda or English) and location (e.g., home or workplace). We compensated participants 16,500 UGX (~$5 USD) for IDI participation.

### Data preparation and analysis

Ugandan qualitative researchers recorded, transcribed verbatim, and translated into English all FGDs and IDIs. Because our primary interest was in peer-delivered HIV prevention interventions, this sub-study focuses exclusively on FGDs and IDIs from the HIVST intervention arms. We conducted thematic analysis of our data using a combination of inductive and deductive coding [19–21]. We foremost utilized deductive codes from a theory of social support that included four dimensions of support: instrumental (provision of tangible goods and services or aides), informational (provision of advice, suggestions, and information), emotional (provision of care, empathy, love, and trust), and appraisal (provision of information for self-evaluation) [22]. This theory was developed to understand structures which influence health outcomes [22–24] and has been used to evaluate HIV PE programs among FSWs in Bangladesh [25] as well as HIV treatment adherence among diverse populations living with HIV (including young women) in Uganda and Kenya [26–28]. Through repeated readings of the data, we also developed inductive codes that captured elements of role modeling, financial support, and peer stigmatization.

Authors MM and JW, both of whom have graduate-level training in qualitative research, developed, revised, and independently applied the codebook to a subset of interviews (*n* = 4), compared their coding, and resolved disagreements via group discussion. The lead author (MM) then coded all IDIs (*n* = 30) and FGDs (*n* = 5) in NVivo version 12 (QSR International, Melbourne, Australia). Authors MM, SDR, SAM, and KFO agreed on themes and mapped these onto the dimensions of social support.

**Fig. 1:**
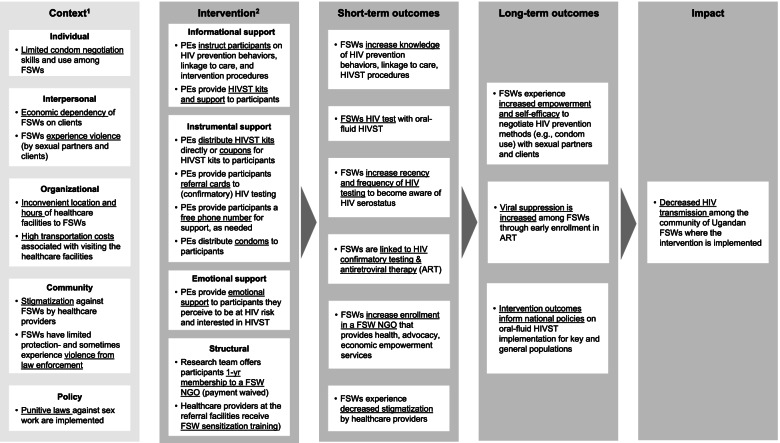
Theory of change for an FSW peer-delivered HIV self-testing intervention. ^1^ Constructs from the Socio-Ecological Model [31] ^2^ Types of social support hypothesized to be provided by FSW peer educators in this intervention

To inform the development of future FSW peer-delivered HIV prevention interventions, we also solicited from the intervention designers, authors DKM, TB, and KFO, information about the kinds of support that were purposefully built into the design of the intervention. The theory of change for how FSW social support is hypothesized to affect the intervention outcomes [29, 30], is depicted in Fig. 1. This figure includes the anticipated short-term (e.g., increased frequency of HIVST), long-term (e.g., increased viral suppression among FSWs who tested HIV-positive and initiated ART), and overall (e.g., decreased HIV transmission among Ugandan FSWs in the intervention setting) anticipated impact of this intervention. We additionally mapped how PEs’ and participants’ experiences with social support in this intervention compared with the intended intervention design and whether these motivated or discouraged HIVST uptake.

## Results

In February 2017, we completed five FGDs with 28 PEs (median age 33 years, interquartile range [IQR] 29–37 years). Between March and April 2017, we completed 30 IDIs with participants randomized to either the direct HIVST delivery arm (*n* = 19) or the coupon exchangeable for HIVST arm (*n* = 11). The median age of participants was 30 years (IQR 27–33 years), most (80%, *n* = 24) reported a primary or secondary level of education, and the majority (74%, *n* = 22) reported testing for HIV in the past year, Table 1.

**Table 1:** The socio-demographic characteristics of IDI participants, (*n* = 30)

Age (median, IQR)	30 (27–33)
Education	
*No formal education*	3 (10%)
*Primary/Junior*	13 (43%)
*Secondary*	11 (37%)
*Vocational*	1 (3%)
*Tertiary*	2 (7%)
Monthly income, UGX^a^	
*< 120,000*	9 (30%)
*120,000–250,000*	4 (13%)
*250,000–500,000*	13 (44%)
*> 500,000-1,000,000*	4 (13%)
Years in sex work (median, IQR)	7.5 (3–11)
Number of clients on an average night (median, IQR)	5 (4–8)
Inconsistent condom use with clients^b^	15 (50%)
Time since last HIV test	
*> 3–6 months*	14 (47%)
*> 6–12 months*	8 (27%)
*> 12–24 months*	3 (10%)
*> 24 months*	3 (10%)
*Never tested*	2 (6%)
Tested HIV-positive (rapid testing)^c^	8 (35%)

^a^Income in Ugandan Shillings (UGX); comparable to United States Dollars (USD) < $35, $35–$75, $75–$150, >$150 using the exchange rate of 3363.85 UGX = 1 USD on October 10th, 2016

^b^ Participants reported condomless sex with at least one client during an average working night

^c^ Represents 23 participants who agreed to blood-based rapid HIV testing at study completion (month 4)

In our data we identified three of the four types of social support: informational, instrumental, and emotional support [22]. We also modified the theory of social support by identifying how each of these types of support either motivated or discouraged participants from accepting the peer-delivered HIVST intervention. Motivating and discouraging social support factors, as experienced by participants, are mapped against the intervention design elements in Table 2**.**

**Table 2:** The influence of FSW social support on peer-delivered interventions: intervention experiences vs. design

Social support domains^**a**^	PE & participant experiences^**b**^	Intervention design^**c**^
**Informational** *The provision of advice, suggestions, and information*	• (+) PEs and participants instructed and assisted each other with HIVST kit use and results interpretation • (+/−) Participants shared with each other positive and negative experiences using the HIVST kit • *(−)*^*d*^ *Some PEs and participants spread misinformation on HIV transmission and the HIVST kit (*e.g.*, product safety)*	• Verbal: PEs provided instruction on HIV prevention behaviors (e.g., condom use, partner selection, family planning options), HIVST procedures, results interpretation, linkage to HIV care • Physical: HIVST kit contained written and pictorial instructions (in Luganda and English) from kit manufacturer on use, results interpretation, and linkage to care
**Instrumental** *The provision of tangible goods and services or aides*	• (+) PEs in the direct-delivery arm distributed HIVST kits directly to participants; some PEs in the coupon-delivery arm redeemed coupons for participants and directly distributed HIVST kits to facilitate testing access (which was not part of the intended intervention design) • (+) PEs and participants supported each other’s access to healthcare services (e.g., pooled financial resources for travel) • *(−)*^*d*^ *HIVST kits were not always available at healthcare facilities (coupon-delivery arm) and fostered mistrust of the PEs delivering the intervention*^*d*^	• PEs delivered to participants HIVST kits (direct-delivery arm) or coupons exchangeable for free HIVST kits at specified, nearby healthcare facilities (coupon-delivery arm) • PEs gave participants a referral card for free HIV testing (referral-only arm) or confirmatory testing (intervention arms) at nearby healthcare facilities • PEs provided participants with a study card containing a free phone number for medical doctors and counsellors, which could be used to facilitate interpretation of HIVST results, linkage to confirmatory testing or treatment, or help with adverse events • PEs distributed free condoms
**Emotional** *The provision of care, empathy, love, and trust*	• (+) Participants felt supported by knowledgeable PEs who acted in their best interests • (+) PEs and study peers supported participants’ HIVST uptake, confirmatory testing, and treatment adherence *(+/−)*^*d*^ *Participants had mixed feelings about disclosure of their HIV status to PEs; some anticipated support (*e.g.*, linkage to care assistance), while others had some concerns (*e.g.*, involuntary HIV status disclosure, social exclusion)* ^*d*^	• The intervention designers assumed that PEs would provide participants with emotional support because PEs were instructed to select individuals for the study whom they perceived to be at HIV risk and thought would be interested in HIVST

^a^Definitions of informational, instrumental, and emotional according to House [22] and Heaney and Israel [24]

^b^ In cases where FSW social support motivates or discourages HIVST uptake, this is indicated with a (+) or (-) respectively

^c^ Intervention design elements were provided by authors: DKM, TB, and KFO

^d^ Cases where contextual factors indirectly impacted social support dimensions

### Informational support: FSW communication networks can facilitate the spread of information

Participants reported being motivated to take up HIVST when PEs provided them with direct informational support (i.e., accurate information on HIV prevention and HIVST). Specifically, participants reported that direct instruction from PEs on how to use the HIVST kit (intended by the intervention design) and their assistance using the kit encouraged them to try HIVST. One participant reported, “I was told [by my PE that] when two lines appear [on the HIVST kit] you are [HIV-] positive and one line you are [HIV-] negative” (direct-delivery arm, age 24). Another participant described how their PE “recorded the minutes for me and when it was due [i.e., when the test was finished], she told me to withdraw it [the swab] from the [buffer] tube” (direct-delivery arm, age 35). This instruction commonly occurred through a vertical exchange of information from PEs to participants. However, in some cases participants instructed each other on HIVST kit use and results interpretation, thereby indicating a horizontal exchange of information:

Some of my colleagues [study peers] called me and asked me to guide them on how to [use the HIV self-] test and I told them exactly how I had done mine and they also followed the same steps. I told them not to eat anything after brushing [their teeth] so that the test is accurate (direct-delivery arm, age 30)

Participants also reported being motivated to HIVST or being able to motivate their study peers to HIVST, by sharing positive HIVST experiences. For example, one participant described, “I offered to share my experience with them [study peers] after testing first. We all shared our results out of excitement for having used this new technology” (coupon-delivery arm, age 29). Participants were also encouraged to accept the intervention after those who were “bold”, and role modeled HIVST ensured that there were no adverse side effects (FGD 4). Conversely, a few participants shared negative HIVST experiences, such as gum discomfort after swabbing, that reportedly caused some participants to hesitate to engage with the intervention (FGD 2).

Some participants were also discouraged from accepting the intervention and/or trusting the test results when they received misinformation about HIV transmission and HIVST from peers in the community, that were not study peers. For example, one participant described how discussions with peers in her community, around the transmission of HIV through saliva made her apprehensive about the accuracy of oral-fluid HIVST kit results: “The more I interact with people who keep saying that saliva has no HIV, the more I get some doubts about it” (coupon-delivery arm, age 29). Other participants hesitated to accept HIVST because of misinformation they received from study peers that called into question the safety of the HIVST kit and/or the integrity of the intervention implementers:

[Participants] were worried, asking questions like, “Why are they [the study implementers] only involving sex workers?” [And saying] that it seems the [HIVST] kits are poisoned, and they intend to kill sex workers [and] that they have put [in] a virus that kills people slowly by slowly … (FGD 5)

Other PEs described how some participants shared misinformation about side effects following HIVST use, such as causing “cancer and diseases” (FGD 2) or making one “paralyzed in the mouth” (FGD 4).

### Instrumental support: pooling resources influences the uptake of HIVST and healthcare services

Per the intervention design, PEs and participants often helped one another access, uptake, and utilize healthcare services. In fact, some PEs in the coupon-delivery arm described helping peers in their group overcome burdens associated with having to visit healthcare facilities to access HIVST kits (e.g., time and financial constraints) by redeeming participants’ HIVST coupons on their behalf (thus, mirroring the direct-delivery HIVST arm).

Additionally, participants in both intervention arms pooled resources to mitigate financial barriers (e.g., transportation costs) to linkage to care. One participant described, “We [peers] communicate a lot, and sometimes when we have a friend who is sick, we collect money and take her to the hospital” (coupon-delivery arm, age 20). Financial support was also reported between PEs and their participants when one PE described, “I get my money and tell her [a participant] to go and get treatment [antiretroviral therapy; ART]” (FGD 2).

While instrumental support often motivated HIVST uptake, product stock outs at healthcare facilities (i.e., limited HIVST kit availability) indirectly discouraged participants from accepting the intervention. Such contextual factors resulted in participants in the coupon-delivery arm expressing mistrust and frustration towards their PEs when they were “getting to the clinics and [were] not being given the HIV self-test kits” (FGD 4). One PE in the coupon-delivery arm also described how limited HIVST kit availability at healthcare facilities strained her relationship with participants to whom she had promised to obtain an HIVST kit: “Most of the clinics had not got the [HIVST] kits by the time we [PEs] went there. This caused trouble between me and the participants” (FGD 2).

### Emotional support: FSWs experienced support or social exclusion from peers

Participants described being more inclined to accept an HIVST intervention when delivered by knowledgeable PEs who they believed had their best interests in mind and who provided reassurance that using the HIVST kit “doesn’t have any side effects” (FGD 1). For example, one participant reported, “I didn’t have any [concerns before taking the HIVST] because I trust my PE. She is a friend of mine, and I know for sure that she can’t allow something bad to be introduced to us” (direct-delivery arm, age 40). In addition, several participants emphasized the importance of confidentiality and reported disclosing their HIV status to trusted PEs rather than “other sex workers” (direct-delivery arm, age 28) who may not keep their HIV status private.

Furthermore, participants described being motivated to accept HIVST kits when study peers provided emotional support by traveling together to healthcare facilities to exchange coupons for HIVST kits (coupon-delivery arm) and when study peers encouraged confirmatory HIV testing in addition to initiating HIV treatment.

Finally, participants described anticipated and actual outcomes of HIV status disclosure following HIVST as either motivating or discouraging them to engage in HIV prevention or treatment interventions. For example, after disclosing her HIV status to her PE, one participant reported that her PE encouraged her to “keep protecting myself [against HIV]” (direct-delivery arm, age 31). Similarly, PEs often encouraged participants who tested HIV-positive to seek treatment: “I told her … be strong [and] go to the health facility (ART clinic)” (FGD 2).

Although many participants experienced emotional support as intended by the intervention design, fear of involuntary HIV status disclosure and anticipated stigmatization from FSW communities in which the intervention was delivered, discouraged some participants from accepting the intervention. Some participants were discouraged by anticipated stigmatization following involuntary HIV-positive status disclosure, while others feared social exclusion following involuntary HIV-negative status disclosure because they perceived most of their peers to be living with HIV. One participant who tested HIV-negative described fearing that, upon learning her negative status, peers living with HIV would not call on her to receive “sugar and clothes” and would discourage her from sitting with them in a bar (direct-delivery, age 28). To mitigate these challenges, another participant said: “[I] pretend I am [HIV-] positive. I have to pretend I am like them [my peers]” (direct-delivery arm, age 27).

## Discussion

Ugandan FSWs in this study supported each other informationally, instrumentally, and emotionally to engage in HIVST. While these forms of social support mostly encouraged intervention uptake and linkage to care, contextual factors and their indirect influence on social support sometimes discouraged FSWs from engaging in HIVST. FSWs felt motivated to accept HIVST when their PE provided accurate intervention information, directly delivered HIVST kits, and acted in their best interest, as well as when they felt emotionally supported by their PEs and study peers. In contrast, FSWs hesitated to use peer-delivered HIVST or coupons for HIVST when they heard negative experiences and misinformation about HIVST from peers (in the study and in their communities), when HIVST availability was limited due to product stock outs, and when they feared negative social consequences from their communities following involuntary HIV status disclosure.

These findings highlight the complexity of utilizing word-of-mouth peer systems to disseminate intervention information, thereby emphasizing the need for intervention evaluations. While peer instruction on HIVST use was intended by the intervention, direct peer assistance conducting HIVST (e.g., timing, and interpreting HIVST results) was not intended and may pose risk of testing coercion or involuntary HIV status disclosure [15]. Furthermore, word-of-mouth peer networks were also prone to the quick spread of misinformation surrounding HIV risk and HIVST. While some of this misinformation around HIVST was shared by FSWs not enrolled in the study, we also found that misinformation shared between study peers persisted after PE intervention visits and was often only resolved through the sharing of positive HIVST experiences or role modeling. This indicates that while the intervention was experienced as successful regarding its distribution of verbal and pictorial HIVST information, some FSWs may benefit from having PEs or peers demonstrate HIVST use on themselves (i.e., role modeling) to mitigate fears surrounding adverse effects of HIVST. Thus, we recommend that future interventions enhance the informational support shared between study peers through role modeling, while maintaining awareness of the context in which the intervention is delivered and the misinformation which can be shared between FSW peers in the community. For example, future studies may utilize “champion” PEs (also referred to as “mentor mothers” in Ugandan prevention of mother-to-child transmission programs) who have had success with HIV prevention interventions, to share their experiences, and to address any concerns or misinformation about intervention side effects [32, 33]. We also recommend that future interventions intermittently evaluate the information being shared between FSW peers and adjust informational support accordingly to mitigate the spread of misinformation.

Furthermore, findings from this study emphasize the barriers Ugandan FSWs face while accessing facility-based care and the advantages of direct peer-delivered interventions. Like other FSW studies in this setting, [34] the PEs and peers in our study overcame barriers to intervention use by pooling resources (e.g., HIVST coupons, travel funds). In some cases, such assistance resulted in participants in the coupon-delivery arm receiving HIVST kits directly from their PEs, thus likening this intervention arm to the direct-delivery arm and indicating a general preference among Ugandan FSWs for direct delivery over coupon-delivery, a preference that was also confirmed in findings from the larger implementation trial [4]. We also speculate that PEs may have redeemed coupons to gain trust from their participants, remain well liked, and mitigate jealousy among participants in different study arms. Based on these findings, we recommend the direct peer delivery of future HIV prevention interventions, as this may better meet FSW preferences and further promote intervention uptake. Additionally, future FSW peer-delivered interventions should consider the delivery of multiple versus single intervention units (e.g., providing multiple HIVST kits at a time) to increase intervention penetration within the community, as demonstrated successfully among Ugandan men who have sex with men [3].

The findings that peer emotional support was a primary incentive to HIVST uptake among FSWs supported underlying assumptions in the intended intervention design. This research highlights the importance of who the PE is and the type of support they provide. PEs who are knowledgeable, maintain confidentiality of the peer’s HIV status, and provide reassurance should be recruited for future peer-delivered interventions and can be successfully identified by collaborating with established peer network organizations (including FSW NGOs) within the community [25]. Future interventions should collaborate with these selected peer educators at early stages of intervention development to ensure intervention appropriateness in the target population. Additionally, to enhance the advantages of peer emotional support, future peer-delivered interventions might incorporate the concept of an intervention partner (i.e., an individual to whom FSWs can be accountable or who can hold them accountable), which has been demonstrated to increase HIV pre-exposure prophylaxis (PrEP) and ART adherence in similar settings [32, 35, 36].

Our research finally recognizes a unique balance between the emotional support required for intervention uptake and the fear of social consequences from communities in which the intervention is delivered (e.g., social exclusion following involuntary HIV status disclosure) resulting from intervention uptake. While previous literature indicates stigmatization and social exclusion from FSW peer groups living and not living with HIV, we did not expect to find some participants misreporting an HIV-negative result for fear of social exclusion [16, 34]. The perception held among FSWs that most peers are living with HIV merits further investigation to ensure it does not dissuade FSWs from regular HIV testing or misreporting testing results.

While these findings provide novel insight into the role of social support on peer-delivered HIVST uptake, this study has some limitations. First, since we recruited participants through PEs who often worked within established, hierarchical structures (e.g., guest houses), FSWs who were not part of these structures (and were likely of lower social standing) may not have been recruited and thus may be underrepresented in this study [2, 37]. Second, HIVST misinformation may have emerged as a major theme, in large part, because HIVST was not commercially available in Uganda outside of research settings at the time this study was conducted. Finally, because this study used secondary data from a larger randomized HIVST trial that did not design the FGD or IDI guides with the dimensions of social support in mind, our findings may have overlooked some aspects of social support relevant to peer-delivered HIV interventions among FSWs [4, 38].

## Conclusion

This qualitative study highlights how social support (e.g., informational, instrumental, and emotional support) among Ugandan FSW peers can work in ways that both motivate and discourage peer-delivered intervention uptake. Based on our findings, we suggest that future FSW peer-delivered HIV prevention interventions first work with community organizations to identify trusted and knowledgeable PEs, train these individuals on how to successfully deliver the intervention, and give them the opportunity to develop their own experiences with the intervention before having them directly deliver the intervention to their peers. We also encourage future interventions to consider the context in which the FSW peer-delivered intervention will be delivered and to develop strategies to mitigate anticipated challenges (e.g., community-level misinformation, product unavailability, and community-level stigmatization). These approaches may help maximize the uptake and impact of future peer-delivered HIV prevention interventions in Uganda and other similar settings.

## Data Availability

All quantitative data (e.g., questionnaires and descriptive statistics) are made publicly available by the International Initiative for Impact Evaluation (3ie): https://dataverse.harvard.edu/dataset.xhtml?persistentId=doi:10.7910/DVN/OVEAC9. Qualitative data used in this study is not publicly available but is available upon reasonable request.

## Abbreviations

ART: Antiretroviral therapy
FGD: Focus group discussion
FSW: Female sex worker
HIVST: HIV self-testing
IDI: In-depth interview
IQR: Interquartile range
MARPI: Most at Risk Population Initiative
NGO: Non-governmental organization
PE: Peer educator
PrEP: Pre-exposure prophylaxis
UGX: Ugandan Shillings
USD: United States Dollars
